# Supplementary material for: Comparative multiomics analysis of cell physiological state after culture in a basket bioreactor
Source: Sci Rep. 2022 Nov 23;12:20161. doi: 10.1038/s41598-022-24687-4 (PMC9686226; doi:10.1038/s41598-022-24687-4)
Supplement: Supplementary file 1 — Supplementary Information 1. [file 41598_2022_24687_MOESM1_ESM.zip › raw data/Transcriptomic raw data/6.Enrichment/1.GO/TE1vsTC1/GSEA/TC1/CHEMOKINE_RECEPTOR_BINDING/CHEMOKINE_RECEPTOR_BINDING(GO_0042379).html]

Details for gene set CHEMOKINE\_RECEPTOR\_BINDING(GO:0042379)[GSEA]


|  || Dataset | readcount.sample |
| Phenotype | sample.cls |
| Upregulated in class | TC1 |
| GeneSet | CHEMOKINE\_RECEPTOR\_BINDING(GO:0042379) |
| Enrichment Score (ES) | -0.24657185 |
| Normalized Enrichment Score (NES) | -1.0 |
| Nominal p-value | 0.0 |
| FDR q-value | 1.0 |
| FWER p-Value | 1.0 |
Table: GSEA Results Summary

  

Fig 1: Enrichment plot: CHEMOKINE\_RECEPTOR\_BINDING(GO:0042379)
  
Profile of the Running ES Score & Positions of GeneSet Members on the Rank Ordered List

  

| PROBE | DESCRIPTION   (from dataset) | GENE SYMBOL | GENE\_TITLE | RANK IN GENE LIST | RANK METRIC SCORE | RUNNING ES | CORE ENRICHMENT || 1 | 103242767 | LOC103242767 |  |  | 597 | Infinity | -0.0258 | No |
| 2 | 103235798 | LOC103235798 |  |  | 2528 | 1.846 | 0.0505 | No |
| 3 | 103233831 | CCL25 |  |  | 9178 | 1.074 | -0.1520 | Yes |
| 4 | 103215781 | CXCL12 |  |  | 10760 | 0.972 | -0.1371 | Yes |
| 5 | 119624339 | LOC119624339 |  |  | 11035 | 0.954 | -0.0653 | Yes |
| 6 | 103244577 | CXCL14 |  |  | 11809 | 0.906 | -0.0201 | Yes |
| 7 | 103219246 | CCL21 |  |  | 13181 | 0.821 | -0.0090 | Yes |
| 8 | 103235823 | CXCL11 |  |  | 15365 | 0.685 | -0.0463 | Yes |
| 9 | 103242769 | LOC103242769 |  |  | 15693 | 0.662 | -0.0026 | Yes |
| 10 | 103235786 | CXCL8 |  |  | 16435 | 0.610 | 0.0180 | Yes |
| 11 | 103219254 | CCL27 |  |  | 17363 | 0.544 | 0.0244 | Yes |
| 12 | 103215218 | CCL28 |  |  | 18005 | 0.489 | 0.0388 | Yes |
| 13 | 103235787 | CXCL6 |  |  | 18442 | 0.446 | 0.0586 | Yes |
| 14 | 103242710 | CCL2 |  |  | 19585 | 0.330 | 0.0366 | Yes |
| 15 | 103235789 | CXCL1 |  |  | 19654 | 0.321 | 0.0618 | Yes |
| 16 | 103235824 | CXCL10 |  |  | 20200 | 0.241 | 0.0587 | Yes |
| 17 | 103218000 | CCL20 |  |  | 20517 | 0.187 | 0.0610 | Yes |
| 18 | 103242750 | CCL5 |  |  | 20742 | 0.140 | 0.0634 | Yes |
| 19 | 103242711 | CCL7 |  |  | 20831 | 0.121 | 0.0701 | Yes |
| 20 | 103233059 | CCL17 |  |  | 21092 | 0.000 | 0.0585 | Yes |
| 21 | 103246650 | CCL24 |  |  | 21994 | 0.000 | 0.0182 | Yes |
Table: GSEA details
[plain text format]

  

Fig 2: CHEMOKINE\_RECEPTOR\_BINDING(GO:0042379)
  
Blue-Pink O' Gram in the Space of the Analyzed GeneSet

  

Fig 3: CHEMOKINE\_RECEPTOR\_BINDING(GO:0042379): Random ES distribution
  
Gene set null distribution of ES for
 **CHEMOKINE\_RECEPTOR\_BINDING(GO:0042379)**
